# Ezetimibe Versus Alirocumab and Plaque Vulnerability in Patients with Acute Coronary Syndrome Not Achieving LDL-Cholesterol Target with Statin Therapy: The COMBI-LLT-ACS Randomized Trial

**DOI:** 10.3390/jcm15197564

**Published:** 2026-09-29

**Authors:** Anna Kovalskaya, Guzel Bikbaeva, Dmitry Duplyakov, Polina Duplyakova, Ekaterina Sukhinina, Ekaterina Savinova

**Affiliations:** 1 Department of Propedeutic Therapy with Course of Cardiology, Samara State Medical University, Samara 443099, Russia; duplyakov@yahoo.com; 2 V. Polyakov Samara Regional Clinical Cardiological Dispensary, Samara 443070, Russia; guzelbikbaeva63@mail.ru (G.B.); polina_duplyakova@yahoo.com (P.D.); sukhinina@samsmu.ru (E.S.); caterinacomarowa@yandex.ru (E.S.)

**Keywords:** acute coronary syndrome, lipid-lowering therapy, vulnerable plaque, myocardial infarction, statins, low-density lipoprotein cholesterol, percutaneous coronary intervention, biomarkers, Ezetimibe, Alirocumab

## Abstract

**Objective**: To compare two lipid-lowering therapy strategies involving Ezetimibe and Alirocumab on the vulnerability of atherosclerotic plaque (AP) in patients with acute coronary syndrome (ACS) not achieving the LDL-cholesterol target while on intensive statin therapy. **Methods**: Combi-LLT-ACS (Patients receiving combination lipid-lowering therapy admitted with the acute coronary syndrome) included 125 patients aged 59 (51; 64) years, of whom 67.2% were men. All underwent percutaneous coronary intervention of the IRA; also, all patients had at least one stenosis < 50% in non-IRA, had high compliance with statins, and did not reached LDL-C level of ≤1.4 mmol/L. Patients underwent Coronary Computed Tomography Angiography (CCTA) to detect vulnerable AP as well as lipid profile and numerous biomarkers: neutrophil to lymphocyte ratio (NLR), monocyte to lymphocyte ratio (MLR), monocyte to high-density lipoprotein ratio (MHR), systemic inflammatory response index (SIRI), matrix metalloprotease type 9 (MMP-9), tissue inhibitor of metalloproteinases type 1 (TIMP-1), Galectin-3 (GAL-3), neutrophil gelatinase-associated lipocalin (NGAL), and C-reactive protein (CRP). Thereafter, they were randomized into two groups, Ezetimibe vs. Alirocumab, on the same visit. The follow-up period was 52 weeks. We used the following CCTA criteria to detect vulnerable AP: positive remodeling, napkin-ring sign, spotty calcification, and low-attenuation plaque. **Results**: One month after ACS, vulnerable APs were observed by CCTA in 56 patients (44.8%). After 52 weeks, 10 (17.9%) patients showed stabilization of previously vulnerable APs or a decrease in the number of AP vulnerability criteria. At week 52, TC and LDL-C levels decreased significantly over time in both groups. Both arms of therapy demonstrated a positive effect on the inflammatory markers and markers of extracellular matrix remodeling. Direct comparison of changes between groups revealed no statistically significant differences in the number of vulnerability criteria (*p* = 0.930); however, in the group receiving Ezetimibe, we found a statistically significant change over the observation period (*p* = 0.032). **Conclusions**: Among patients with ACS, receiving a high-intensity statin in combination with Ezetimibe or Alirocumab prevented the emergence of new vulnerable coronary lesions over 52 weeks. While direct regression of specific vulnerability criteria was not observed, these therapeutic strategies were associated with a reduction in LDL-C and biomarkers of inflammation and extracellular matrix remodeling.

## 1. Introduction

Significant advances have been made in the management of acute coronary syndrome (ACS), driven by both the widespread implementation of invasive procedures and the use of highly effective drug therapy regimens. Current versions of both Russian and European clinical guidelines regulating the management of patients with ACS recommend combination lipid-lowering therapy (LLT) in patients who have had a myocardial infarction (MI) but failed to achieve target low-density lipoprotein (LDL) levels on high-dose statin monotherapy. The second recommended component may be Ezetimibe or a proprotein convertase subtilisin-kexin type 9 (PCSK9) inhibitor, which have proven their efficacy and safety in a number of randomized clinical trials (RCTs) [1,2,3,4,5]. More recent RCTs confirmed stabilization of vulnerable atherosclerotic plaques (AP) by adding PCSK9 inhibitors to basic statin therapy, mainly by reducing their volume and increasing the thickness of the fibrous cap [6,7,8,9,10,11,12,13,14]. However, none of these studies compared two LLT regimens directly (statin + Ezetimibe vs. statin + PCSK9 inhibitors) in terms of simultaneous stabilization of both biomarkers and imaging vulnerability criteria, along with the achievement of LDL-C targets. Given the expanding indications for the early administration of LLT, a direct comparison of these treatment regimens and their role in the stabilization of vulnerable AP, mediated by a reduction of chronic inflammation, in a properly designed prospective RCT is relevant. Against this background, we aimed to assess the efficacy (markers of atherosclerotic plaque vulnerability) and safety of Ezetimibe versus Alirocumab in patients presenting with acute coronary syndromes and not achieving the LDL-cholesterol target while on intensive statin therapy on a randomized basis.

## 2. Materials and Methods

### 2.1. Study Design

**Figure 1:**
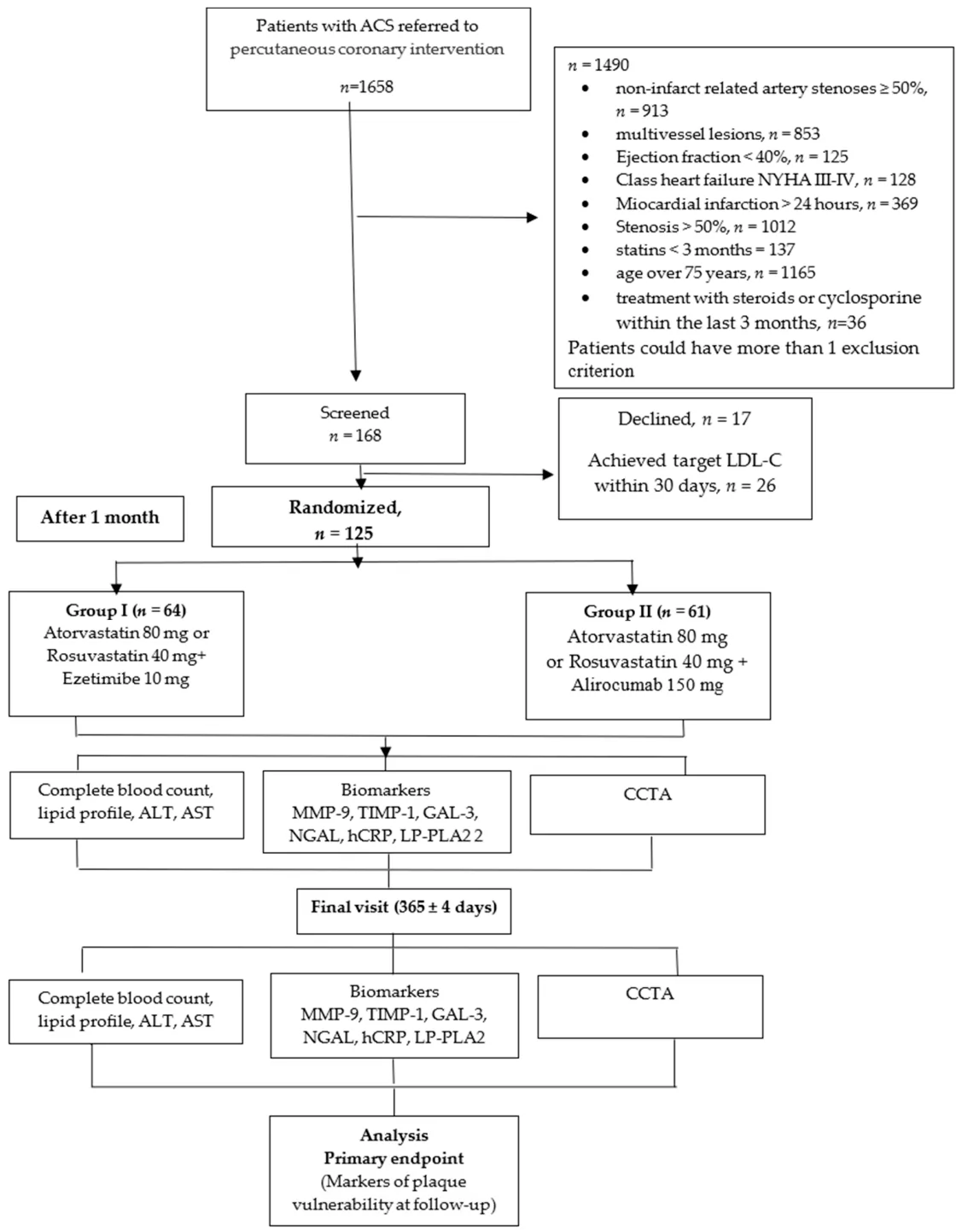
Study flowchart. ACS—acute coronary syndrome, CCTA—Coronary Computed Tomography Angiography, LDL-C—low-density lipoprotein, HDL-C—high-density lipoprotein; LP-PLA2—lipoprotein-associated phospholipase A2, ALT—alanine aminotranspherase, AST—aspartate aminotranspherase, CK—creatine kinase, NLR—neutrophil to lymphocyte ratio, MLR—monocyte to lymphocyte ratio, Mon-HDL-C—monocyte tohigh-density lipoprotein ratio, SIRI—systemic inflammatory response index, MMP-9—matrix metalloprotease type 9, TIMP-1—tissue inhibitor of metalloproteinases type 1, Gal-3—Galectin-3, NGAL—neutrophil gelatinase-associated lipocalin, CRP—C-reactive protein.

This open-label, prospective, randomized, single-center study included 125 patients admitted to a tertiary center of the ACS network (Figure 1). Patients were enrolled in the study sequentially. The study was approved by the Ethics Committee of the Samara State Medical University of the Ministry of Health of the Russian Federation (Protocol No. 253 dated 14 September 2022). The institutional order regarding preparations for the study was signed on 1 October 2022, while patient enrollment itself began on 10 November 2022 (with an outpatient visit taking place one month after the inpatient stay. The study was registered on ClinicalTrials.gov (NCT05624658) with no funding sources; the protocol adheres to the CONSORT guidelines. The protocol of the Combi-LLT-ACS study has been published previously [6].

### 2.2. Participants

Inclusion criteria: age 18–75 years; acute coronary syndrome (unstable angina (UA) or MI) with at least one coronary artery stenosis requiring percutaneous coronary intervention (PCI); MI duration up to 24 h; AP in one or two non-infarction-related coronary arteries with <50% stenosis; no statin use for <3 months before the index ACS; not achieving LDL-cholesterol target on admission; failure to achieve the target LDL-C level ≤ 1.4 mmol/L at the second visit; signed informed consent.

Exclusion criteria: previous MI; history of revascularization (PCI or coronary artery bypass grafting); stenosis of a non-infarction-related artery ≥ 50%; multivessel or left main disease; EF < 40%; Killip III–IV; chronic heart failure III–IV NYHA; significant calcification or tortuosity of the coronary arteries; severe renal and hepatic insufficiency; allergic reactions to iodine-containing contrast agents; intolerance to statins, aspirin, P2Y12 blockers; patients who previously received PCSK9 inhibitors and/or Ezetimibe; treatment with systemic steroids or systemic cyclosporine within the last 3 months; inflammatory diseases; cancer within the last 5 years; planned any surgical intervention within 3 months; severe mental disorders; pregnancy or breastfeeding period.

The total follow-up period was 52 weeks. At the final visit (365 ± 4 days), patients again underwent CCTA and an assessment of the same laboratory parameters. All enrolled patients completed the study; there were no early discontinuations due to intolerance, adverse events, or withdrawal of consent. The primary analysis was conducted according to the intention-to-treat (ITT) principle. The ITT population included all 125 patients who underwent randomization, regardless of treatment adherence. No patients withdrew from the study early, and all 125 patients completed the 12-month follow-up and had baseline and final CCTA data. Therefore, all randomized patients were included in the analysis of the primary endpoint.

No major deviations from the approved study protocol were recorded. Only minor deviations from the planned procedure timing were noted. In three patients, the control CCTA study was performed approximately ±4 days later than the scheduled date due to personal reasons. Additionally, in three patients, Alirocumab administration was performed approximately ±3 days later than the scheduled date due to logistical reasons. These deviations did not result in exclusion of patients from the ITT population and did not prevent the protocol-specified analysis.

There were no treatment crossovers: no patients crossed over from the Ezetimibe group to the Alirocumab group or vice versa during the observation period.

All 125 randomized patients had complete pairs of CCTA studies (baseline and 12-month assessments), ensuring 100% availability of CCTA data for analysis.

### 2.3. Randomization

All patients underwent PCI of the infarct-related artery with drug-eluting stents. In-hospital patients were on guideline-directed medical therapy according to the Russian clinical guidelines [2,3]. Statins were prescribed initially at the maximum dose (atorvastatin 80 mg/d or rosuvastatin 40 mg/d).

At the second visit (30 ± 2 days), those who demonstrated high compliance and did not achieve target LDL-C levels (≤1.4 mmol/L) were randomized into two groups. Participants were assigned to intervention groups using random assignment. Randomization was performed using a computer-generated random sequence to assign participants in a 1:1 ratio to both groups. The allocation sequence was computer-generated using the random number function in Microsoft Excel.

Patient enrollment was performed by study investigators, whereas the allocation sequence was maintained by a designated study member who was independent of patient enrollment and postoperative outcome assessment.

Group 1—Ezetimibe (10 mg/day) was added on top of statin therapy (64 patients, M, 65.1%, mean age 60 years). Group 2—subcutaneous biweekly injection of Alirocumab at a dose of 150 mg on top of statin therapy (61 patients, M, 70.5%, mean age 59 years). Randomized patients underwent CCTA and thorough laboratory testing the same day.

The randomization procedure was not disclosed to any of the investigators or the study coordinator.

### 2.4. Primary Outcome

Primary endpoint: decrease or absence of increase in the number of vulnerability criteria by CCTA in non-infarct-related coronary arteries.

CCTA (for detection of vulnerable atherosclerotic plaques) was performed on a 128-slice GE Revolution EVO system with ECG synchronization and intravenous contrast with 100 mL of the iodine-containing radiocontrast agent “Iogexol”. The coronary calcium index was determined using the Agatston method. The vulnerability criteria (positive remodeling, napkin-ring sign, spotty calcification, and low attenuation plaque) of atherosclerotic plaque were assessed in the Plaque ID, SmartScore 4.0, Ver.: “SS4.0_203” software sequentially by two experienced radiologists with a 90 ± 4.5% agreement, separately for each of the three main epicardial coronary arteries (left anterior interventricular branch, left circumflex artery, and right coronary artery). The assessments were performed independently and completely blinded to patient clinical data, treatment group assignment (Ezetimibe/Alirocumab), and the results of previous assessments for the same patient. The radiologists were also blinded to the time sequence of the studies; that is, when assessing the images, they were not informed whether a particular study was performed at baseline or post-treatment.

To minimize the potential influence of previous assessments, each set of images was assessed independently of the results from other time points.

In addition to the previously reported percentage agreement, we quantitatively assessed interobserver reproducibility. Cohen’s kappa coefficients with 95% confidence intervals were calculated for categorical vulnerability criteria:

Low-attenuation plaque: κ = 0.82 (95% CI: 0.76–0.88);

Positive remodeling: κ = 0.79 (95% CI: 0.72–0.86);

Spotty calcifications: κ = 0.75 (95% CI: 0.68–0.82).

For quantitative parameters, including plaque volume and stenosis degree, the intraclass correlation coefficient (ICC) was calculated and found to be 0.94 (95% CI: 0.91–0.96).

Thus, the obtained values demonstrate a high degree of interobserver agreement for categorical characteristics and high reproducibility of quantitative measurements.

In accordance with the definition of the primary endpoint, the primary level of statistical analysis was patient-level analysis. A patient was classified as having a vulnerable lesion if at least one coronary artery contained a plaque that met at least one of the predefined vulnerability criteria.

### 2.5. Secondary Outcomes

Secondary endpoints: lipid profile dynamics (comparison of baseline and final values in each treatment group), biomarker dynamics (comparison of baseline and final values in each treatment group), mortality from all causes and from cardiovascular diseases, stent thrombosis/restenosis, non-fatal myocardial infarction/unstable angina, emergency revascularization (PCI).

Some hematological indices were derived from complete blood count—neutrophil–lymphocyte ratio (NLR), platelet–lymphocyte ratio (PLR), monocyte–lymphocyte ratio (MLR), monocyte to high-density lipoprotein (HDL-C) ratio Mon/HDL-C, systemic immune inflammation index (SII), and systemic inflammatory response index (SIRI). Complete lipid profile, alanine aminotransferase (ALT), aspartate aminotransferase (AST), hs-Troponin I, Galectin-3, hs-C-reactive protein (hs-CRP), matrix metalloproteinase-9 (MMP-9), tissue inhibitor of metalloproteinase type 1 (TIMP-1), lipocalin associated with neutrophil gelatinase (NGAL), and lipoprotein-associated phospholipase A2 (LP-PLA2) were also assessed.

Prespecified secondary endpoints: Markers of inflammation and extracellular matrix remodeling, such as MMP-9, TIMP-1, Galectin-3, NGAL, and high-sensitivity CRP, were included in the original protocol and registration form.

Exploratory biomarkers: Hematological indices NLR, MLR, SIRI, and Mon/HDL-C were added during the study, as they are available markers of systemic inflammation and have been described in many studies.

Laboratory parameters assessment: (1) A complete blood count was performed using the standard method with a white blood cell differential on a Mindray BC-6200 analyzer (Guangzhou, China). The following parameters were assessed: hemoglobin, WBC, platelet, and absolute lymphocyte, neutrophil, and monocyte counts. These parameters were used to calculate the following:

NLR—neutrophil-to-lymphocyte ratio;

PLR—platelet-to-lymphocyte ratio;

MLR—monocyte-to-lymphocyte ratio;

Mon/HDL—monocyte-to-HDL cholesterol ratio;

SII—(neutrophil count × platelet count) ÷ (lymphocyte count);

SIRI—(neutrophil count × monocyte count) ÷ (lymphocyte count).

Blood biochemistry was performed on a Beckman Coulter AU680 automated biochemical analyzer (Brea, CA, USA) with the appropriate test systems for assessing the lipid profile (TC, LDL-C, HDL-C, TG), CPK, ALT, and AST.

The level of MMP-9 (detection range 0.156–10 ng/mL, sensitivity 0.055 ng/mL), TIMP-1 (detection range 0.156–10 ng/mL, sensitivity 0.055 ng/mL), Galectin-3 (detection range 0.156–10 ng/mL, sensitivity 0.054 ng/mL), NGAL (detection range 0.156–10 ng/mL, sensitivity 0.065 ng/mL) was determined by direct enzyme immunoassay using the Human ELISA Kit (Cloud-Clone Corp, Wuhan, China) on a Multiskan FC Thermo Fisher Scientific apparatus (USA). Blood for the study was taken on an empty stomach in vacuum tubes with a coagulation activator and gel, kept for 30 min at room temperature; after centrifugation for 15 min at 2500× *g* rpm, the blood serum was frozen at −27 °C (up to 3 weeks), then transported in batches for storage in a freezer at −70 °C and subjected to ELISA as biosamples accumulated.

Outcome analysis was performed through independent assessment by the Clinical Events Committee.

### 2.6. Statistical Analysis

Statistical analysis was performed by SPSS Statistics v. 27, Past (v. 4.17), and GraphPad Prism10 (GraphPadSoftware, Inc.). The sample calculation was performed (based on the PACMAN-MI study) using a two-sided test at a significance level of α = 0.05 and a statistical power of 1 − β = 0.80 (80%), which allowed the inclusion of 125 patients in the study. Based on the expected between-group treatment effect and variability derived from the available imaging data, the calculation yielded approximately 53 evaluable patients per treatment group, corresponding to 106 patients in total. To account for a prespecified 15% attrition/non-evaluable imaging rate, the required recruitment target was calculated as: 106/(1 − 0.15) = 125 and was therefore rounded up to 125 patients. A total of 125 patients were randomized, with 61 patients allocated to one treatment group and 64 to the other.

Continuous variables were described using the median and interquartile range, while qualitative variables were described using absolute and relative frequencies. Differences between the two groups for a quantitative variable were examined using the Mann–Whitney U test. To compare two groups for a qualitative variable, the Pearson χ^2^ test was used for expected frequencies > 5; in other cases, Fisher’s exact test with Monte Carlo simulation was used (*n* = 99,999). Qualitative variable frequencies in multi-field tables were compared using Pearson’s χ^2^ test, provided that >80% of the contingency table cells had expected frequencies ≥ 5 and none had frequencies < 1. If these conditions were violated, Fisher’s exact test with Monte Carlo simulation (*n* = 99,999) was used; for subsequent pairwise comparisons, Fisher’s exact test with a Bonferroni correction was used. To examine differences between two dependent groups by quantitative variable, the paired Wilcoxon signed-rank test (W) was used; for qualitative variables, the McNemar test was used. To compare three or more dependent groups by quantitative variable, the Friedman test was used; post hoc analysis was performed using the Wilcoxon signed-rank test. For binary variables (vulnerability criteria based on CCTA data), we used contingency tables to determine the odds ratio (OR) and 95% confidence interval. An analysis of covariance (ANCOVA) was also performed. The Bonferroni correction was applied for multiple comparisons. Survival analysis was performed using the Kaplan–Meier method; the log-rank test was used to compare two curves. The results were considered statistically significant at *p* < 0.05.

## 3. Results

The Combi-LLT-ACS, open-label, prospective, randomized, single-center study enrolled 125 patients (enrollment period 10 November 2022–24 July 2023). At baseline, both groups were comparable in terms of gender, age, risk factors and comorbidities, as well as the type of ACS (Table 1) and main baseline laboratory parameters, except for LDL-C level. Therapy at discharge is shown in Table S1. All patients received comparable therapy throughout the study.

CCTA was performed on average one month after ACS, and vulnerable AP were observed in 56 patients (44.8%). The total number of vulnerable AP did not differ between the groups at baseline, and the frequency of all vulnerability parameters was also comparable (Table 2). Comparing the number of vulnerable plaques (with at least one vulnerability criterion) over 12 months in the Ezetimibe group, we found a statistically significant change (*p* = 0.032), despite the median values remaining unchanged.

A statistically significant increase in the calcium score (CAC) was also observed in the Ezetimibe group.

Table 2 shows that the dynamics in the Alirocumab group in terms of the number of criteria and plaques were generally similar to those of Ezetimibe in terms of median values, which indicates that Alirocumab is also effective in preventing the appearance of new plaques, but in this study did not show an advantage over Ezetimibe in this specific parameter. Direct comparison of the changes between the groups revealed no statistically significant differences in the number of vulnerability criteria (*p* = 0.930), and no statistically significant between-group differences in the number of vulnerable plaques (*p* = 0.894).

The dynamics of the lipid profile and biochemical parameters characterizing the effectiveness and safety of LLT are presented in Table 3 Compliance with the oral lipid-lowering drug Ezetimibe was assessed every 3 months by counting returned blisters and checking with electronic patient diaries; patients were also contacted by telephone monthly. Treatment adherence was quantified using the medication possession ratio (MPR). The mean MPR for the Ezetimibe group was 96.8 ± 4.1%. Over the 52-week period, the average number of missed injections of Alirocumab was 1.6 ± 1.1 per patient. A missed dose was defined as the absence of a dose (at our lipid center) within 3 days of the scheduled date. In the event of a missed dose, the patient received a follow-up phone call within 24 h. At week 52, TC and LDL-C levels decreased significantly over time in both groups. The study showed that LDL-C levels decreased statistically significantly in both groups. However, over the follow-up period, patients on Alirocumab showed a more significant decrease in LDL-C than patients receiving Ezetimibe—1.33 (1.09; 1.70) vs. 1.49 (1.36; 1.79) mmol/L (h = 0.006). Before initiation of protocol-specified combination lipid-lowering therapy, baseline LDL-C levels were 3.54 (Q1; Q3: 2.78; 4.29) mmol/L in the Ezetimibe group and 3.95 (3.22; 4.75) mmol/L in the Alirocumab group (*p* = 0.017).

After statistical adjustment for baseline LDL-C values, a statistically significant difference was found between groups (F(1109) = 6.39; *p* = 0.013). The adjusted mean LDL-C value at 12 months was 1.61 mmol/L (95% CI: 1.501–1.720) in the Ezetimibe group and 1.41 mmol/L (95% CI: 1.300–1.523) in the Alirocumab group.

We additionally analyzed LDL-C dynamics from the time of randomization (V2) to the end of follow-up (V6). In the Ezetimibe group, the median LDL-C level decreased from 1.96 (1.76–2.44) to 1.49 (1.36–1.79) mmol/L (*p* < 0.001), while in the Alirocumab group, it decreased from 2.35 (2.08–2.92) to 1.33 (1.09–1.70) mmol/L (*p* = 0.001). Between-group comparison of LDL-C changes revealed a statistically significant difference (*p* < 0.001), with a more pronounced reduction in LDL-C in the Alirocumab group.

Altogether, 34 patients (56%) on Alirocumab achieved LDL-C levels < 1.4 mmol/L vs. only 24 patients (38%) on Ezetimibe (*p* = 0.041), and another 10 patients (16%) on Alirocumab achieved 1.4–1.8 mmol/L vs. 15 patients (23%) on Ezetimibe (*p* = 0.32). Lp (a) showed a significant decrease among patients taking Alirocumab only: 16.69 (6.07; 90.28) vs. 14.89 (5.14–74.40), *p* <0.001. Both lipid-lowering therapy strategies did not result in a significant increase in ALT and AST, as well as creatine phosphokinase (CK).

Both arms of LLT demonstrated a positive effect on the inflammatory markers and markers of extracellular matrix remodeling. After 12 months, a statistically significant reduction was demonstrated for the following hematological indices: NLR, MLR, SIRI, and Mon/HDL-C. MMP-9, Galectin-3, NGAL, and hsCRP also decreased in both groups, regardless of the drug administered (Table S2). Due to the multiple biomarkers analyzed in Table S2 a Bonferroni correction was applied to control for Type I error inflation across the 10 comparisons within this domain. The adjusted level of significance was set at *p* < 0.005. After Bonferroni correction (*p* < 0.005), the reduction in biomarker levels remained statistically significant in both treatment groups. NLR, MLR, and SIRI did not show significant changes after correction for multiple comparisons.

Given the limited sample size (*n* = 125) and short follow-up period, this study was underpowered to detect differences in major clinical endpoints (MACE). Observed rates were low in both groups, with only 5 events reported (MI—1 event, unstable angina—3 events, Stroke—1 event). Therefore, comparison of clinical outcomes remains preliminary.

There were no significant differences between the two groups in the incidence of adverse reactions (Quincke’s edema, Local allergic reactions, Diarrhea/flatulence, Myalgia) related to the medications used.

## 4. Discussion

Lipid-lowering therapy is a mandatory component for patients diagnosed with acute coronary syndrome (myocardial infarction or unstable angina). Its efficacy and safety have been proven in numerous RCTs and are currently beyond any doubt. Statins are the most studied class of lipid-lowering drugs, and both atorvastatin and rosuvastatin in high doses are most commonly prescribed to patients with ACS. Clinical guidelines for the treatment of patients with ACS, as well as those with dyslipidemia, recommend two or more lipid-lowering drugs if LDL-C targets are not achieved while taking high-intensity statins [1,2,3,4,5].

Until recently, the most commonly used combination was a statin with Ezetimibe, which allowed for an additional 15% reduction in LDL-C levels compared to solo statin therapy [4]. The advent of monoclonal antibodies, namely PCSK9 inhibitors, blocking the protein that controls the expression of LDL-C receptors on the surface of hepatocytes, has made it possible to use this new combination in patients having very high cardiovascular risk [1,2,3,4,5,7].

The RUTHERFORD-2 study evaluated the efficacy of adding Evolocumab on top of statin therapy in patients with familial heterozygous hypercholesterolemia. This trial showed that Evolocumab reduced LDL-C levels by an additional 60% compared with the statin-only group, enabling 67% of patients in the Evolocumab group to achieve the target LDL-C level, compared with 2% among those receiving statin alone [8].

Alirocumab, according to the ODYSSEY phase II and III RCTs, also demonstrated a significant advantage in reducing LDL-C levels over other LLTs. After 24 weeks, the mean reduction in LDL-C levels compared to the baseline was 50.6 ± 1.4% for Alirocumab and 20.7 ± 1.9% for Ezetimibe (*p* < 0.0001). In the same study, 77.0% of patients receiving Alirocumab and 45.6% of patients receiving Ezetimibe achieved LDL-C levels < 1.8 mmol/L (*p* < 0.0001) [9,10].

The PACMAN-AMI study enrolled patients with acute MI who received rosuvastatin and Alirocumab vs. placebo. At baseline, the mean LDL-C level was 152.8 (33.8) mg/dL, and at the final visit it was 74.4 (30.5) mg/dL in the placebo group and 23.6 (23.8) mg/dL in the Alirocumab group (*p* < 0.001). Thus, in the placebo group, the LDL-C level decreased by 76.5 mg/dL compared with the baseline level, and in the Alirocumab group by 131.2 mg/dL (*p* < 0.001). In addition, triglycerides, lipoprotein (a), and apolipoprotein B decreased more significantly in the Alirocumab group [11].

The key goal of any LLT in patients after ACS is not only to achieve target LDL-C, but stabilization of vulnerable plaques. According to a meta-analysis that recruited 9 RCTs and 8 cohort studies with 5607 patients altogether, combination therapy (statin + PCSK9 inhibitor), compared with statin monotherapy, allows achieving LDL-C levels ≤ 1.4 mmol/L in a higher proportion of patients (OR: 5.83; 95% CI: 5.20–6.55) [12].

Gao F et al. showed that the absolute reduction in LDL-C was significantly higher in patients receiving Alirocumab compared with a standard statin therapy (1.72 ± 0.51 vs. 0.96 ± 0.59, *p* < 0.0001). The addition of Alirocumab was associated with a greater increase in the minimum thickness of the fibrous cap of the atherosclerotic plaques (18.0 [10.8–29.2] μm vs. 13.2 [7.4–18.6] μm; *p* = 0.029) and a decrease in the maximum lipid arc as measured by optical coherence tomography (15.1 [7.8–24.5] versus 8.4 [2.0–10.5]; *p* = 0.008) [13].

Nicholls, S.J. et al. demonstrated that Evolocumab resulted in a greater regression in the percentage of atheroma volume compared to placebo (−2.29% ± 0.47% vs. −0.61% ± 0.46%; *p* = 0.009 [14].

In the ARCHITECT study, patients who received Alirocumab in addition to high-dose statin therapy (both in combination with Ezetimibe and without it), the volume of AP by CCTA decreased from 34.6% [32.5–36.8] to 30.4% [27.4–33.4], *p* < 0.001 after 78 weeks. A change in the morphology of AP was also detected: an increase in calcification (+0.3%; *p* < 0.001) and thickness (+6.2%; *p* < 0.001) of the fibrous cap, which indicates stabilization of the atherosclerotic process [15].

The PRECISE-IVUS study evaluated the effects of combination therapy with Ezetimibe and Atorvastatin compared with Atorvastatin monotherapy on the regression of AP, where, in addition to a greater reduction of LDL-C levels, a decrease in the percentage of atheroma volume (78% vs. 58%; *p* = 0.004) and total atheroma volume (75% vs. 58%; *p* = 0.02) was observed [16].

According to our results, only 17% of patients who survived ACS achieved target LDL-C levels (<1.4 mmol/L) within 1 month with high-intensity statin only, necessitating intensification of LLT. The degree of TC and LDL-C reduction among patients taking Alirocumab in addition to a statin was statistically significantly greater than in the statin/Ezetimibe group. Furthermore, the absence of significant changes in laboratory parameters characterizing the safety of LLT also indicates that its intensification does not pose a risk to liver function and muscle tissue. At week 52, the statin/Alirocumab combination showed a greater decrease in TC, LDL-C and Lp (a) than the statin/Ezetimibe combination. Fifty-six percent of patients in the Alirocumab group achieved target LDL-C values, while 38% did so in the Ezetimibe group.

Inflammation is known to be a key pathogenetic mechanism in the development and progression of atherosclerosis, from the early onset to the development of the first clinical manifestations and subsequent cardiovascular complications. In our study, regardless of the LLT type, all markers of inflammation and extracellular matrix remodeling (NLR, MLR, SIRI, Mon/HDL, MMP-9, Galectin-3, NGAL, and hsCRP) were statistically significantly reduced.

In both treatment groups, we observed statistically significant reductions in blood biomarkers. It should be noted that our study did not include a control group receiving high-dose statin monotherapy, as the objective of the study was to compare two treatment strategies. Given the natural resolution of inflammation after ACS, we cannot rule out that some of the observed biomarker changes are due to regression of the acute post-infarction response, rather than a specific pleiotropic effect of Ezetimibe or Alirocumab.

Comparing the number of vulnerable plaques (with at least one vulnerability criterion) over 12 months in the Ezetimibe group, we found a statistically significant change (*p* = 0.032), despite the median values remaining unchanged. This result may indicate no significant increase in the number of vulnerable plaques and a stabilization of plaque formation during the observation period. However, this result does not allow us to conclude that vulnerable plaques completely disappeared or regressed in most patients.

A statistically significant increase in the calcification index (CAC) was also observed in the Ezetimibe group.

This may be related to the natural progression of atherosclerosis (the accumulation of calcium within the plaque structure). The fact that the number of soft vulnerable components (PR, LAP) decreased or remained unchanged may also indicate a beneficial effect: plaques become denser and potentially less prone to rupture [17].

The dynamics in the Alirocumab group in terms of the number of criteria and plaques were generally similar to those in the Ezetimibe group in terms of median values, indicating the effectiveness of Alirocumab in preventing the occurrence of new plaques, but in this study, it did not show advantages over Ezetimibe in this specific parameter. Direct comparison of changes between groups revealed no statistically significant differences in the number of vulnerability criteria (*p* = 0.930) or statistically significant between-group differences in the number of vulnerable plaques (*p* = 0.894).

Despite higher LDL-C levels in the Alirocumab group at randomization, treatment with Alirocumab resulted in a more pronounced reduction in LDL-C levels by the end of the follow-up period compared with Ezetimibe.

We observed a more pronounced reduction in LDL-C levels and a lower final LDL-C level in the Alirocumab group. However, no statistically significant advantage of Alirocumab was observed for the primary CT angiography endpoint—change in the number of vulnerable plaques per patient (*p* = 0.930). Similarly, there were no between-group differences in the change in the number of vulnerable plaques (*p* = 0.894).

Thus, in our study, a discrepancy was observed between the degree of LDL-C reduction and the dynamics of the CT angiographic parameters studied. Possible explanations include the relatively short follow-up period and small patient sample, as well as the complex and multifactorial nature of the structural reorganization of atherosclerotic plaques.

While our study was not designed to assess cellular phenotypes, the observed trends are biologically plausible. One potential mechanism is the direct modulation of macrophage polarization by Ezetimibe—specifically, inhibition of the NF-κB/iNOS pathway and promotion of an M2 reparative phenotype within the plaque [14]. However, as we did not perform immunohistochemistry or transcriptomic analysis in this trial, this remains a hypothesis requiring validation in future translational studies.

## 5. Conclusions

Among patients with ACS, receiving high-intensity statin in combination with Ezetimibe or Alirocumab prevented the emergence of new vulnerable coronary lesions over 52 weeks. While direct regression of specific vulnerability criteria was not observed, these therapeutic strategies were associated with a reduction in LDL-C and biomarkers of inflammation and extracellular matrix remodeling.

### Study Limitations

- The study was conducted at a single center;
- The study was randomized and included less than 5% of the total number of patients with ACS admitted to our center;
- Relatively small sample size;
- Baseline LDL-C imbalance;
- Insufficient statistical power for clinical events

## Figures and Tables

**Table 1:** Baseline characteristics.

Parameters	Ezetimibe (*n* = 64)	Alirocumab (*n* = 61)	All Patients (*n* = 125)	*p*
Men, *n* (%)	42 (65.1%)	43 (70.5%)	85 (68%)	0.560
Women, *n* (%)	22 (34.9%)	18 (29.5%)	40 (32.3%)	
Age, yrs, Me (Q_1_; Q_3_)	60 (51; 66)	59 (50; 63)	59 (51; 64)	0.395
BMI, kg/m^2^, Me (Q_1_; Q_3_)	28.2 (25.5–30.9)	27.4 (25.4–31.3)	27.7 (25.5–31.1)	0.648
Smoking, *n* (%)	28 (43.8%)	29 (47.5%)	57 (45.6%)	0.671
Hypertension, *n* (%)	61 (95.3%)	60 (98.4%)	121 (96.8%)	0.619
CKD				
-GFR ≥ 90 mL/min/1.73 m^2^, *n* (%)	9 (14.1%)	13 (21.3%)	22 (17.6%)	0.391
-GFR 60–89 mL/min/1.73 m^2^, *n* (%)	41 (64.1%)	40 (65.6%)	81 (64.8%)	
-GFR 45–59 mL/min/1.73 m ^2^, *n* (%)	10 (15.6%)	7 (11.5%)	17 (13.6%)	
-GFR 30–44 mL/min/1.73 m ^2^, *n* (%)	2 (3.1%)	1 (1.6%)	3 (2.4%)	
-Diabetes mellitus, *n* (%)	10 (15.6%)	4 (6.6%)	14 (11.2%)	0.155
Type of ACS				
Unstable angina, *n* (%)	15 (23.4%)	14 (23.0%)	29 (23.2%)	0.923
NSTEMI, *n* (%)	22 (34.4%)	23 (37.7%)	45 (36.0%)	
STEMI, *n* (%)	27 (42.2%)	24 (39.3%)	51 (40.8%)	
Total cholesterol, mmol/L	5.51 (4.74; 6.39)	6.07 (4.93; 7.1)	5.77 (4.83; 6.65)	0.119
LDL-C, mmol/Λ	3.54 (2.78; 4.29)	3.95 (3.22; 4.75)	3.82 (3.05; 4.51)	**0.017**
Triglycerides, mmol/L	1.24 (0.84; 2.04)	1.31 (0.79; 2.11)	1.26 (0.84; 2.08)	0.619
HDL-C, mmol/Λ	1.27 (0.99; 1.54)	1.26 (1.06; 1.53)	1.26 (1.03; 1.53)	0.771
Troponin I, ng/mL Me (Q_1_; Q_3_)	4.3 (2.7; 7.0)	5.6 (3.3; 8.9)	4.6 (2.9; 7.4)	0.130
LP-PLA2,ng/mL Me (Q_1_; Q_3_)	78.6 (63.4; 139.1)	80.6 (65.0; 126.8)	80.6 (64.4; 132.9)	0.617
Lp (a), mg/L, Me (Q_1_; Q_3_)	13.55 (5.73; 34.89)	16.69 (6.07; 90.28)	14.78 (5.87; 48.29)	0.358

BMI—body mass index; GFR—glomerular filtration rate; CKD—chronic kidney disease; NSTEMI—non-ST-elevation MI; STEMI—ST-elevation MI; LDL-C—low-density lipoprotein; HDL-C—high-density lipoprotein; LP-PLA2—lipoprotein-associated phospholipase A2; Lp (a)—lipoprotein a.

**Table 2:** CCTA baseline and final parameters.

Parameters, Me (Q_1_; Q_3_)	Ezetimibe (*n* = 64)			Alirocumab (*n* = 61)			OR [95%CI]	*p*
Positive remodeling, *n*	22 (34.4%)			21 (34.4%)			0.96 [0.46–1.99]	1.000
Low attenuation plaque, *n*	15 (23.4%)			14 (23%)			0.94 [0.41–2.15]	0.886
Spotty calcifications, *n*	9 (34.4%)			6			0.64 [0.21–1.93]	0.428
Napkin-Ring Sign, *n*	1 (1.6%)			1 (1.6%)			1.03 [0.06–16.90]	1.000
Positive remodeling at final visit, *n*	18 (28%)			17 (27.8%)			0.95 [0.43–2.06]	0.893
Low attenuation plaque at final visit, *n*	15 (23.4%)			14 (23%)			0.91 [0.40–2.10]	0.832
Spotty calcifications at final visit, *n*	6 (9.4%)			4 (6.6%)			0.64 [0.17–2.40]	0.509
Napkin-Ring Sign at final visit, *n*	1 (1.6%)			1 (1.6%)			0.64 [0.21–1.93]	0.428
**Parameters,** **Me (Q_1_; Q** **_3_** **)**	**E** **zetimibe** **(** ***n*** **= 64** **)**			**Alirocumab** **(*n* = 61)**			**Visit 2** **Between Groups**	
	**Visit 2**	**Visit 6**	***p***	**Visit 2**	**Visit 6**	***p***	***p***	
CAC (Agatston units)	62 (16; 217)	69 (17; 207)	**0.018** 7 [95%CI 1–13]	61 (1; 239)	62 (0; 245)	0.935	0.605	
Number of Vulnerability Criteria per patient, *n*	0 (0; 2)	0 (0; 1)	**0.016** −0.45 [95%CI 0.82–0.08]	0 (0; 2)	0 (0; 2)	0.314	0.930	
Number of vulnerable AP per patient, *n*	0 (0; 1)	0 (0; 1)	**0.032** −0.15 [95%CI −0.35–0.00]	0 (0; 2)	0 (0; 1)	1.0	0.894	

AP—atherosclerotic plaque.

**Table 3:** Lipids and biochemical parameters at baseline and on final visit.

Parameters Me (Q_1_; Q_3_)	Ezetimibe (*n* = 64)			Alirocumab (*n* = 61)			Visit 2 Between Group	Visit 6 Between Group
	Visit 2	Visit 6	*p*	Visit 2	Visit 6	*p*	*p*	*p*
Total cholesterol, mmol/L	3.40 (3.03; 4.01)	3.00 (2.58; 3.34)	<0.001	4.07 (3.41; 4.71)	2.74 (2.41; 3.20)	<0.001	<0.001	0.063
LDL-C, mmol/L	1.96 (1.76; 2.44)	1.49 (1.36; 1.79)	<0.001	2.35 (2.08; 2.92)	1.33 (1.09; 1.70)	0.001	<0.001	0.006
Triglycerides, mmol/L	1.29 (0.95; 1.78)	0.95 (0.72; 1.45)	<0.001	1.24 (0.93; 1.89)	0.97 (0.75; 1.62)	<0.001	0.874	0.537
HDL-C, mmol/L	1.10 (0.95; 1.26)	1.19 (1.01; 1.43)	0.003	1.15 (0.96; 1.34)	1.19 (1.04; 1.44)	0.056	0.074	0.460
ALT, U/L	27.7 (17.0; 43.4)	28.5 (19.4; 38.0)	0.874	26.2 (21.5; 39.3)	24.4 (18.9; 32.4)	0.138	0.837	0.288
AST, U/L	22.7 (19.3; 29.2)	26.3 (22.1; 32.5)	0.033	22.7 (19.4; 28.9)	25.7 (21.5; 30.8)	0.096	0.859	0.591
CK, U/L	113.4 (77.4; 157.0)	110.4 (91.4; 146.5)	0.175	116.3 (88.4; 171.0)	131.2 (92.4; 220.1)	0.019	0.618	0.094
Lp (a), mg/L	13.55 (5.73; 34.89)	12.14 (5.56; 34.89)	0.136	16.69 (6.07; 90.28)	14.89 (5.14; 74.40)	<0.001	0.322	0.630
LP-PLA2, ng/mL	78.6 (63.4; 139.1)	60.0 (40.2; 106.6)	<0.001	80.6 (65.0; 126.8)	66.6 (43.0; 108.8)	<0.001	0.617	0.978

LDL-C—cholesterol of low-density lipoprotein, HDL-C—cholesterol of high-density lipoprotein, ALT—alanine aminotransferase, AST—aspartate aminotransferase, CK—creatine kinase, Lp (a)—lipoprotein a, LP-PLA2—lipoprotein-associated phospholipase A2.

## Data Availability

The de-identified datasets generated and/or analyzed during the current study are not publicly available due to privacy and ethical restrictions regarding patient confidentiality. The data supporting the findings of this study are available from the corresponding author upon reasonable request.

## Supplementary Materials

The following supporting information can be downloaded at: https://www.mdpi.com/article/10.3390/jcm15197564/s1, Table S1: Pharmacological therapy received during the study; Table S2: Dynamics of biomarkers after 52 weeks FU.
