# Supplementary material for: Ezetimibe Versus Alirocumab and Plaque Vulnerability in Patients with Acute Coronary Syndrome Not Achieving LDL-Cholesterol Target with Statin Therapy: The COMBI-LLT-ACS Randomized Trial
Source: J Clin Med. 2026 Sep 29;15(19):7564. doi: 10.3390/jcm15197564 (PMC13665717; doi:10.3390/jcm15197564)
Supplement: Supplementary file 1 [file jcm-15-07564-s001.zip › jcm-4515705-supplementary.pdf]

Table S1. Pharmacological therapy received during the study.

Medications	Ezetimibe (n=64)	Alirocumab (n=61)	All patients (n=125)	P
Atorvastatin, n (%)	62 (96,9%)	56 (91,8%)	118 (94,4%)	0,266
Rosuvastatin, n (%)	2 (3,1%)	5 (8,2%)	7 (5,6%)	
ACE inhibitors/ARB, n (%)	63 (98,4%)	58 (95,1%)	121 (96,8%)	0,287
Beta blockers, n (%)	64 (100%)	60 (98,4%)	124 (99,2%)	0,488
Acetylsalicylic acid, n (%)	61 (95,3%)	59 (96,7%)	120 (96,0%)	1,0
Clopidogrel, n (%)	20 (31,2%)	17 (27,9%)	37 (29,6%)	0,679
Ticagrelor, n (%)	44 (68,8%)	44 (72,1%)	88 (70,4%)	

ACE inhibitors - inhibitors of angiotensin converting enzyme, ARB - angiotensinIIReceptorBlocker.

Table S2. Dynamics of biomarkers after 52 weeks FU.

Parameter	Ezetimibe (n=64)		Alirocumab (n=61)		P	
	Visit2	Visit 6	Visit 2	Visit 6	Ezetimibe	Alirocumab
NLR	2,26 (1,74;2,98)	2,07 (1,55;2,81)	2,35 (1,87;2,84)	2,06 (1,7;2,68)	**0,026**	**0,026**
MLR	0,25 (0,20;0,33)	0,22 (0,18;0,28)	0,25 (0,20;0,33)	0,24 (0,18;0,31)	**<0,001**	0,051
SIRI	1,18 (0,89;1,61)	0,92 (0,68;1,49)	1,3 (0,95;1,89)	1 (0,71;1,5)	**0,004**	**0,003**
Mon/HDL-C	0,47 (0,34;0,63)	0,41 (0,29; 0,47)	0,47 (0,34;0,65)	0,38 (0,32; 0,53)	**<0,001**	**0,001**
MMP-9, ng/ml	61,38 (33,45;86,92)	32,86 (23,34;41,52)	62,5 (33,85;87,51)	32,77 (25,9;39,95)	**<0,001**	**<0,001**
MMP-9/TIMP-1	1,84 (1,5;2,62)	1,11 (0,69;1,72)	1,87 (1,53;2,96)	0,96 (0,43;1,55)	**<0,001**	**<0,001**
Gal-3, ng/ml	27,21 (4,78;47,27)	3,21 (2,26;5,75)	18,04 (7,43;60,8)	4,7 (1,9;6,52)	**<0,001**	**<0,001**
NGAL, ng/ml	529,67 (293,05;832,13)	351,45 (217,52;486,99)	463,9 (311,61;839,41)	376,22 (258,05;661,4)	**0,005**	**0,036**
hCRP, ng/ml	2,66 (1,67;4,61)	1,09 (0,79;1,98)	2,95 (1,19;6,71)	1,57 (0,94;4,19)	**<0,001**	**0,002**
Troponin I, ng/ml	7,55 (3,80;14,40)	4,30 (2,73;7,03)	8,10 (4,25;13,75)	5,60 (3,33;8,85)	**<0,001**	**<0,001**

NLR - neutrophil to lymphocyte ratio, MLR - monocyte to lymphocyte ratio, Mon-HDL-C - monocyte tohigh-density lipoprotein ratio, SIRI - systemic inflammatory response index, MMP-9 - matrix metalloprotease type 9, TIMP-1 - tissue inhibitor of metalloproteinases type 1, Gal-3 - galectin-3, NGAL - neutrophil gelatinase-associated lipocalin, CRP - C-reactive protein.
